# Preparation of F-doped H_2_Ti_3_O_7_-{104} nanorods with oxygen vacancies using TiOF_2_ as precursor and its photocatalytic degradation activity

**DOI:** 10.1039/d1ra07329j

**Published:** 2021-11-01

**Authors:** Yue Jian, Huayang Liu, Jiaming Zhu, Yaqiong Zeng, Zuohua Liu, Chentao Hou, Shihua Pu

**Affiliations:** Chongqing Academy of Animal Sciences Chongqing 402460 China pu88962@126.com; College of Geology and Environment, Xi'an University of Science and Technology Xi'an 710054 China 807484470@qq.com; Scientific Observation and Experiment Station of Livestock Equipment Engineering in Southwest, Ministry of Agriculture and Rural Affairs Chongqing 402460 China

## Abstract

Photocatalytic degradation is an eco-friendly and sustainable method for the treatment of water pollutants especially tetracycline hydrochloride (TCH). Herein, we developed F-doped H_2_Ti_3_O_7_-{104} nanorods with oxygen vacancies using TiOF_2_ as a precursor by simple alkali hydrothermal and ion-exchange methods. The phase structure, surface composition, optical properties, specific surface areas and charge separation were analysed by a series of measurements. The effects of KOH concentration on the structure and properties of H_2_Ti_3_O_7_ were investigated. It is confirmed that the TiOF_2_/H_2_Ti_3_O_7_ composite can be formed in low concentration KOH solution (1 mol L^−1^), while the H_2_Ti_3_O_7_ single phase can be formed in high concentration KOH solution (>3 mol L^−1^). The prepared F-doped H_2_Ti_3_O_7_-{104} nanorods provide a high specific surface area of 457 m^2^ g^−1^ and a macroporous volume of 0.69 cm^3^ g^−1^. The appropriate mesoporous structure of the photocatalyst makes TCH have a stronger affinity on its surface, which is more conducive to the subsequent photodegradation. Moreover, a synergistic mechanism of photosensitization and ligand–metal charge transfer (LMCT) in the photocatalytic degradation of TCH was proposed. In addition, the prepared F-doped H_2_Ti_3_O_7_-{104} nanorods showed excellent cycle stability and resistance to light corrosion. After five cycles of photodegradation, the degradation rate of TCH was only reduced from 92% to 83%. This low-cost strategy could be used for the mass production of efficient photocatalysts, which can be used for TCH clean-up in wastewater treatment.

## Abstract 2

Efficient photocatalytic degradation of tetracycline hydrochloride by F-doped H_2_Ti_3_O_7_-{104} nanorods.

## Graphical abstract

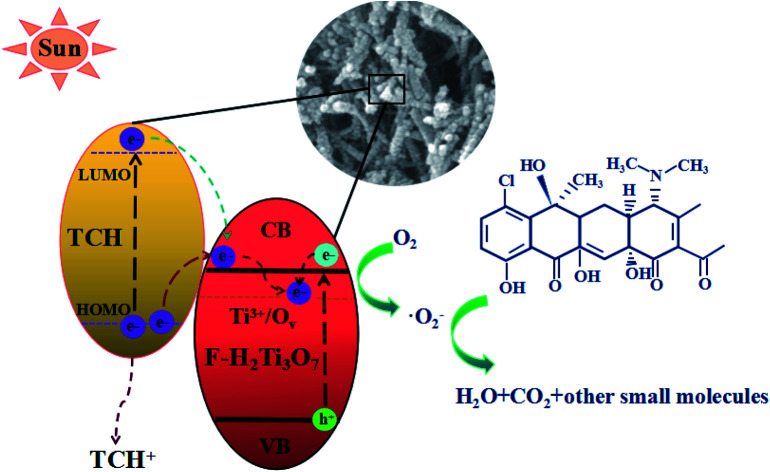

## 1. Introduction

With the widespread use of antibiotics, they have become a new pollutant in the water environment in recent years. It is particularly worth mentioning that more than dozens of sulfonamides, tetracyclines, and fluoroquinolone antibiotics have been detected in surface water and groundwater.^1,2^ Their presence in the aquatic system enhances bacterial resistance, thereby inhibiting the effectiveness of common antibiotics in the treatment of microbial infections.^3–5^

Tetracycline hydrochloride (TCH) is a typical tetracycline broad-spectrum antibiotic, which is widely used as a growth promoter in the treatment of human diseases and animal feeding.^6^ However, TCH is not easily degraded in the environment and poses a huge threat to the ecological environment, aquatic plants, animals, and human health.^7,8^ In order to solve these problems, many advanced technologies have emerged in the field of TCH degradation, such as adsorption,^9^ biodegradation,^10^ electrochemical oxidation,^11^ membrane filtration,^12^ and photocatalytic degradation.^13^ Among them photocatalytic degradation is the most advantageous method to remove total organic carbon because of its low cost, strong oxidizing ability, and being eco-friendly. However, whether the photocatalytic technology can successfully achieve high-efficiency removal of pollutants in the water environment depends on the mineralization capacity and catalytic activity of the photocatalyst.^14,15^ Therefore, the design and synthesis of photocatalysts with excellent mineralization ability and efficient charge separation are of great value to the photodegradation of TCH in water environments.

Hydrogen trititanate (H_2_Ti_3_O_7_), as a new type of titanium-based semiconductor material, has attracted great interest due to its exciting characteristics, such as high specific surface area, high photo-reactivity and non-toxicity. Nowadays, H_2_Ti_3_O_7_ has been used by some researchers for water decomposition,^16^ CO_2_ adsorption,^17^ supercapacitors,^18^ and photocatalytic degradation of organic pollutants.^19–21^ However, a pure H_2_Ti_3_O_7_ only responds to ultraviolet light, and due to the rapid combination of carriers after excitation, the photocatalytic degradation of organic pollutants is not satisfactory.

In recent years, people have conducted a lot of research on improving the photocatalytic activity of H_2_Ti_3_O_7_. High temperature heat treatment, non-metal doping (such as carbon, nitrogen and fluorine) and metal doping/deposition (including transition metals and precious metals) are widely used.^22,23^ In particular, the doping of foreign elements plays a key role in the transient capture of photo-induced electrons and holes, which may be beneficial to inhibit the recombination of photo-generated charge carriers. Non-metallic doping is favored by researchers because of its low cost and simple operation. Fluorine doping can usually generate oxygen vacancies in titanium-based semiconductors by forming Ti–F–Ti bonds.^24^ The appearance of Ti^3+^ is usually accompanied by oxygen vacancies.^25^ This effectively reduces the recombination of carriers. It is worth noting that the remaining unsaturated Ti(vi) atoms may form an electronic coupling with the π orbital connected to –OH in TCH to form a complex of H_2_Ti_3_O_7_ and tetracycline hydrochloride.^26,27^ The strong ligand–metal charge transfer (LMCT) and photosensitization enhance H_2_Ti_3_O_7_ response to visible light.

Nowadays, the most common method is to synthesize Na_2_Ti_3_O_7_ by alkaline hydrothermal method with Ti-based material as the precursor and NaOH as the promoter, and then obtain H_2_Ti_3_O_7_ by the H^+^ exchange method. The reaction conditions play a crucial role in controlling the morphology and size of the H_2_Ti_3_O_7_ nanostructures. For instance, Chang *et al.*^28^ synthesized H_2_Ti_3_O_7_ nanowires directly on titanium foil through a simple alkaline hydrothermal and ion-exchange process. Xiao *et al.*^29^ prepared H_2_Ti_3_O_7_ nanobelts by alkaline hydrothermal and HCl ion-exchange with Degussa P25 as precursor and NaOH as stripping agent. However, most of the synthesized H_2_Ti_3_O_7_ has exposed {020}, {202}, {200} crystal planes, and almost few research has synthesized H_2_Ti_3_O_7_ with {104} crystal plane exposed. TiOF_2_ is a metastable phase, and they often appear as intermediates in the preparation of TiO_2_ with exposed {001} planes.^30,31^ Interestingly, TiOF_2_ can be transformed into F-doped TiO_2_ under certain hydrothermal conditions.^32–35^ Therefore, it is possible to synthesize K_2_Ti_3_O_7_ under alkaline hydrothermal conditions with TiOF_2_ as the source of titanium and fluorine, and KOH as the promoter. Through simple HCl ion-exchange, K_2_Ti_3_O_7_ is further transformed into F-doped H_2_Ti_3_O_7_ nanorods with oxygen vacancies. As far as we know, few people use TiOF_2_ as a precursor to synthesize H_2_Ti_3_O_7_ nanorods.

Herein, we reported a method to synthesize F-doped H_2_Ti_3_O_7_-{104} nanorods with oxygen vacancies by alkaline hydrothermal method and ion exchange method using cubic TiOF_2_ as the precursor KOH as the promoter. The mechanism of synergistic effect of LMCT and photosensitization on the degradation of TCH by H_2_Ti_3_O_7_ nanorods was proposed. This study provides a new idea for the preparation of H_2_Ti_3_O_7_ photocatalyst and the degradation of TCH.

## 2. Experimental section

### 2.1. Chemicals

Tetrabutyl titanate (TBOT, A. R.), potassium hydroxide (KOH, A. R.), benzoquinone (BQ), methanol (MT), *tert*-butyl alcohol (*t*-BuOH), hydrochloric acid (HCl, A. R. 38%), glacial acetic acid (CH_3_COOH, A. R.), hydrofluoric acid (HF, A. R.) were purchased from Fuchen Chemical Reagent Factory, Tianjin, China. Tetracycline hydrochloride (TCH, A. R.) were purchased from Aladdin Industrial Corporation (Shanghai, China). Ultrapure water was used as experimental water.

### 2.2. Preparation of samples

TiOF_2_ photocatalyst was synthesized by a simple one-step hydrothermal method using TBOT as titanium source. 40 mL of CH_3_COOH and 20 mL of TBOT were added into 100 mL beaker, stir for 15 min at 26 °C, and obtained mixture was recorded as A. Next, 8 mL of HF was added to A with uniform stirring speed, and then the mixture was stirred at 26 °C for 1 h (HF has strong corrosivity, so attention should be paid to protection when taking it). Following that, the mixture was transferred into the polytetrafluoroethylene and placed in an autoclave and heated at 180 °C for 5 h. The products were collected by high-speed centrifugation and washed with ultrapure water and absolute ethanol for four times. Following that, dried at 60 °C and named TiOF_2_.

F-doped H_2_Ti_3_O_7_-{104} nanorods were synthesized by the alkali hydrothermal method and ion-exchange method using TiOF_2_ as a precursor, KOH as stripping agent, and HCl as an ion exchanger. 0.5 g of TiOF_2_ was mixed with 50 mL 10 M KOH aqueous solution in a 100 mL polytetrafluoroethylene liner stir at 25 °C for 1 h. Then, the polytetrafluoroethylene was placed in a high-pressure reactor and reacted at 150 °C for 4 h. After that, it was cooled to 25 °C, and the product was washed several times with 0.5 M HCl until the pH value of the filtrate become less than 7. Finally, the product was washed with ethanol and ultra-pure water until the pH value of the filtrate reach to neutral. Following, the sample was dried at 60 °C and named 10 M-TiOF_2_. The above process remained unchanged, except adding 1 M, 3 M, 5 M, and 15 M concentrations of KOH respectively and the obtained photocatalysts were named as 1 M-TiOF_2_, 3 M-TiOF_2_, 5 M-TiOF_2_ and 15 M-TiOF_2_.

To study the interaction mechanism between 10 M-TiOF_2_ and TCH, the complex of 10 M-TiOF_2_ and TCH were prepared. 10 M-TiOF_2_ (20 mg) place into 100 mL TCH (20 mg L^−1^), stirred for 60 min under dark conditions, and then froze and dried at −45 °C by vacuum freeze drier (Beijing Boyikang Scientific Instrument Co., Ltd). The obtained samples were recorded as 10 M-TiOF_2_/TCH.

### 2.3. Characterization of the samples

The crystallinity of samples were confirmed by the Powder X-ray diffraction (XRD; Bruker D8 Advance X-ray diffractometer) at 36 kV, 20 mA equipped with a Cu anode X-ray tube (Cu Kα 1 X-rays, *λ* = 1.54056 Å). The morphology and structure of the samples were analysed by scanning electron microscope (SEM, JSM7500F, Japan) and transmission electron microscopic (TEM, JEOL JEM-2100 operated at 200 kV). The distribution of elements in the samples were analysed by energy dispersive spectrometer (EDS) setting on SEM. The N_2_ adsorption/desorption isotherms of the samples were measured by Micromeritics JW-BK122W. The pore size distribution and specific surface area of the samples were analysed by the Brunauer–Emmett–Teller (BET) and Barrett–Joyner–Halenda (BJH) methods at −196 °C. The chemical bonds and surface functional groups of samples were measured by Fourier transform infrared spectroscopy (FTIR, Nicolet IS5 Spectrometer, USA). The Ultraviolet-visible (UV-Vis) diffuse reflectance spectroscopy (DRS) of the samples were measured by UV-Vis spectrophotometer (Shimadzu Japan). The photoluminescence spectrum (PL) was acquired by a fluorescence spectrophotometer (Shimadzu-RF-6000, Japan). The chemical states of the elements in the samples were determined by X-ray photoelectron spectroscopy (XPS, Thermo SCIENTIFIC ESCALAB). The electron paramagnetic resonance (EPR) was recorded by Bruker 300 EPR electron paramagnetic resonance spectrometer.

### 2.4. Photocatalytic experiment

In order to know the degradation of TCH by the prepared material, 20 mg photocatalyst was dispersed into a quartz reaction tube containing 100 mL TCH (20 mg L^−1^). The quartz tube stirred magnetically for 30 min. After the reaction system reached to the equilibrium of adsorption and desorption, the 500 W xenon lamp was turned on. Then, 7 mL of the mixture was taken out every 5 min, and the centrifuged liquid was used to determine the concentration of TCH. The concentration of TCH in the clarified solution was measured by ultraviolet visible spectrophotometer (L5S, Shanghai precision Instrument Co., Ltd) at the absorption wavelength of 357 nm. To measure the recyclability, the used 10 M-TiOF_2_ was recycled after each photodegradation experiment as follows: the used 10 M-TiOF_2_ was centrifuged, washed several times, and dried at 60 °C for next photodegradation. The dosage of sample was changed from 0.01 g to 0.15 g, 0.20 g and 0.25 g to explore the best dosage of photocatalyst for TCH degradation. The initial concentration of TCH was changed from 20 mg L^−1^ to 30 mg L^−1^, 40 mg L^−1^, and 50 mg L^−1^ to explore the effect of different TCH concentrations on photocatalytic degradation. The main active species (˙O_2_^−^, ˙OH, h^+^) in photocatalytic degradation were explored by adding different scavenger agents (benzoquinone (BQ), *tert*-butyl alcohol (*t*-BuOH) and methanol (MT)) in the reaction system.

### 2.5. Photoelectrochemical measurements

Transient photocurrent experiments were measured by an Autolab (PGSTAT204, Netherlands) in a standard three-electrode system. The fabricated photocatalyst films as the working electrodes, a Pt plate electrode used as the reference electrode, an Ag/AgCl (saturated in KCl) applied as the reference electrode and 0.5 mol L^−1^ Na_2_SO_4_ was used as the electrolyte. The irradiation light source was a 500 W Xe lamp. The electrochemical impedance spectroscopy (EIS) tests were conducted in the same configuration. The frequency ranged from 10^−1^ to 10^5^ Hz and the AC amplitude was adjusted to 5 mV. To prepare the working electrode, the photocatalyst (5 mg) and 5 wt% Nafion solution (80 μL) were dispersed in isopropyl alcohol (920 μL). Then, the mixture (100 μL) was drop casted on a 5 cm × 1 cm fluorine-doped tin oxide (FTO) glass electrode.

## 3. Results and discussion

### 3.1. Crystal structure of photocatalysts

**Fig. 1:**
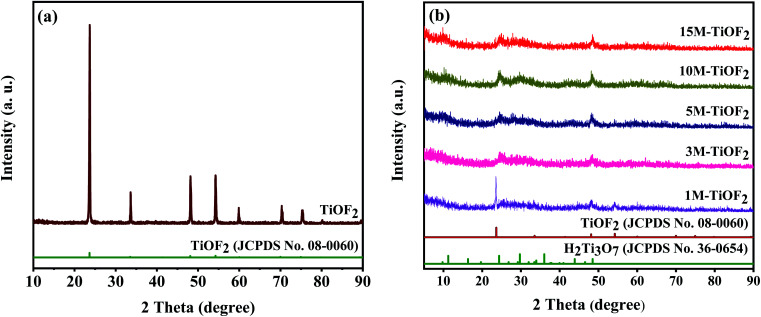
XRD patterns of TiOF_2_ precursor (a) and samples obtained from the TiOF_2_ precursor after alkali hydrothermal and ion-exchange treatment (b).

Fig. 1 provides the XRD patterns of the samples. Fig. 1a clearly shows the crystal structure of the prepared TiOF_2_. The diffraction peaks of the TiOF_2_ are very sharp, which shows that the crystal of the material is good. Besides, the diffraction peaks at 2*θ* = 23.68°, 33.50°, 48.14°, 54.18°, 60.13°, and 66.95° are attributed to the {100}, {110}, {200}, {210}, {211}, and {220} planes of cubic TiOF_2_ (JCPDS no. 08-0060) indicating that the cubic TiOF_2_ was successfully prepared.^24,34^

Fig. 1b shows the XRD patterns of the samples obtained from the TiOF_2_ precursor after alkali hydrothermal and ion-exchange treatment. The diffraction peaks at 2*θ* = 23.68°, 33.50°, 48.14°, 54.18°, 60.13°, and 66.95° are attributed to the {100}, {110}, {200}, {210}, {211}, and {220} planes of cubic TiOF_2_ (JCPDS no. 08-0060), and the diffraction peaks at 2*θ* = 9.78°, 11.25°, 24.35°, 29.73°, 48.49° are attributed to the reflections of the {001}, {100}, {102}, {020} planes of H_2_Ti_3_O_7_ (JCPDS no. 36-0654).^21^ For 1 M-TiOF_2_, it has two crystal phases of TiOF_2_ and H_2_Ti_3_O_7_ which indicates that in the presence of low concentration of KOH, a small part of TiOF_2_ cannot be transformed into K_2_Ti_3_O_7_, which cannot participate in the subsequent ion-exchange reaction, so TiOF_2_/H_2_Ti_3_O_7_ composite is formed. For 3 M-TiOF_2_, 5 M-TiOF_2_, 10 M-TiOF_2_, and 15 M-TiOF_2_, only H_2_Ti_3_O_7_ crystal phase was formed. Therefore, pure H_2_Ti_3_O_7_ photocatalyst could be successfully prepared from TiOF_2_ precursor at high concentration of KOH. It is worth noting that 10 M-TiOF_2_ and 15 M-TiOF_2_ have good crystallinity, indicating that high concentration of KOH is conducive to crystallization.

### 3.2. Morphology analysis of samples

**Fig. 2:**
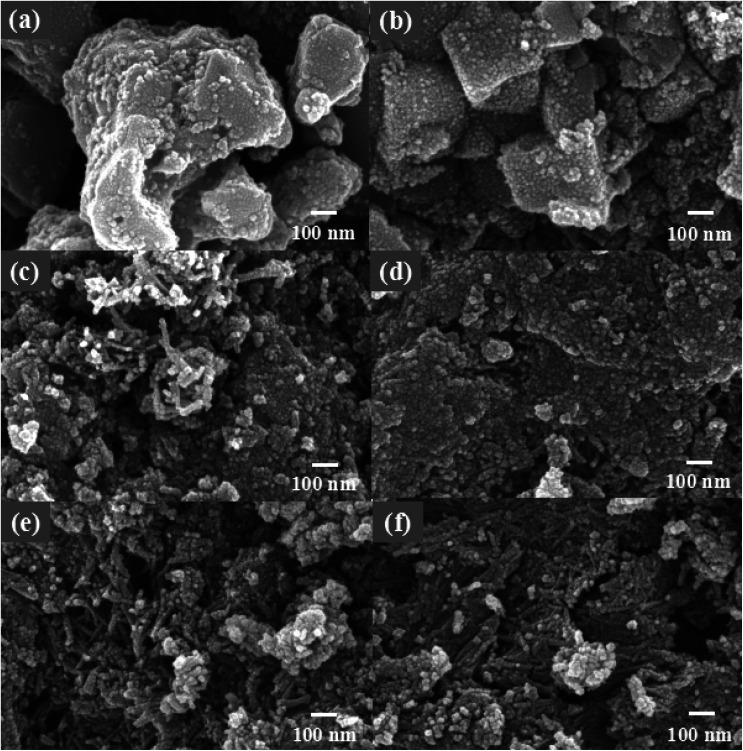
SEM images of (a) TiOF_2_, (b)1 M-TiOF_2_, (c) 3 M-TiOF_2_, (d) 5 M-TiOF_2_, (e) 10 M-TiOF_2_ and (f) 15 M-TiOF_2_.

The morphology of all samples was further studied by scanning electron microscopy analysis. As shown in Fig. 2a, the TiOF_2_ precursor consists of a mixture of cubic and spherical particles with an average size of 280–500 nm (which corresponds to the diameter of the sphere or the side of the cube). Compared with some other results of the prepared TiOF_2_,^35–37^ the morphology of the precursor obtained in this study is not uniform.

Fig. 2b–f show the images of the products obtained by alkaline hydrothermal and ion-exchange using TiOF_2_ as a precursor with different mole of KOH. It can be seen that the morphology of the samples changed greatly after alkali treatment and ion-exchange. In the alkaline hydrothermal environment of 1 M KOH, the surface of TiOF_2_ was etched and the cubic surface became rough (Fig. 2b). This is because part of the cubic TiOF_2_ was stripped by KOH to form K_2_Ti_3_O_7_, and then exchanged with H^+^ to form a small part of H_2_Ti_3_O_7_ single crystal particles with the size of 30–50 nm attached to the surface of the residual cubic TiOF_2_. When the KOH is further increased to 3 M, the phase of TiOF_2_ has completely transformed into H_2_Ti_3_O_7_, and the cubic TiOF_2_ precursor no longer exists (Fig. 2c). 3 M-TiOF_2_ shows that the irregular H_2_Ti_3_O_7_ particles with the size of 20–30 nm aggregate into blocks, and some H_2_Ti_3_O_7_ particles aggregate into rods with the size of 50–100 nm. With the increase of KOH to 5 M, it can be seen that there are a lot of staggered H_2_Ti_3_O_7_ nanorods in 5 M-TiOF_2_, and some H_2_Ti_3_O_7_ particles are aggregated into lamellar structure (Fig. 2d).

When the concentration of KOH is 10 M, H_2_Ti_3_O_7_ nanorods are staggered with an average size of 40–70 nm, and a small part of H_2_Ti_3_O_7_ is aggregated into irregular blocks with an average size of 200–300 nm (Fig. 2e). These nanorods assembled by H_2_Ti_3_O_7_ particles have many reactive sites, and increase the contact area between H_2_Ti_3_O_7_ and TCH, which makes TCH more easily adsorbed on its surface for photocatalytic degradation.^38^ When the concentration of KOH is 15 M, H_2_Ti_3_O_7_ nanorods with an average size of 70–150 nm tend to form. These nanorods are more aggregated, and the pore ratio is less than 10 M-TiOF_2_ (Fig. 2f).

**Fig. 3:**
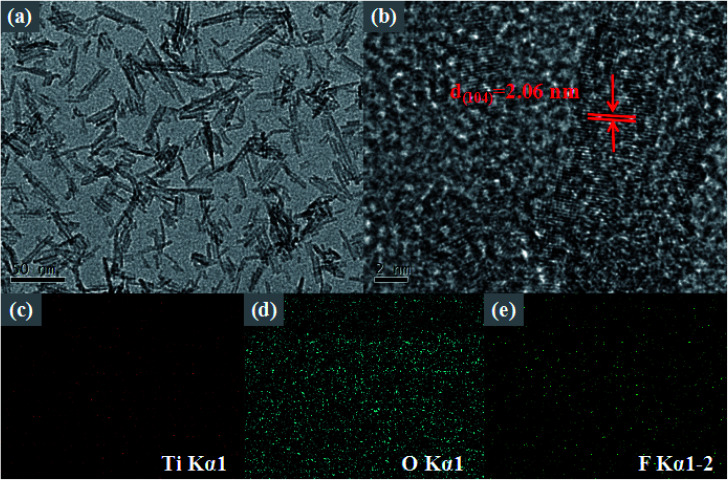
TEM and HRTEM images of 10 M-TiOF_2_ (a and b); elemental mapping of 8-TF (c–e).

To further study the morphological features of the 10 M-TiOF_2_, TEM, HRTEM, and EDS images of prepared 10 M-TiOF_2_ are shown in Fig. 3. It can be seen from Fig. 3a that 10 M-TiOF_2_ is composed of nanorods with an average size of 40–70 nm, which is consistent with the results of SEM observation. In Fig. 3b, the HRTEM image of 10 M-TiOF_2_ nanorods shows a crystal plane spacing of 2.06 nm, which corresponds to the {104} crystal plane of H_2_Ti_3_O_7_. Comparison In order to further describe the element distribution of 10 M-TiOF_2_, an element mapping analysis was performed on 10 M-TiOF_2_ (Fig. 3c–e). Ti, O, and F are evenly distributed. These results confirm that we have successfully synthesized F-doped H_2_Ti_3_O_7_-{104} nanorods.

### 3.3. BET specific surface area and pore structure

**Fig. 4:**
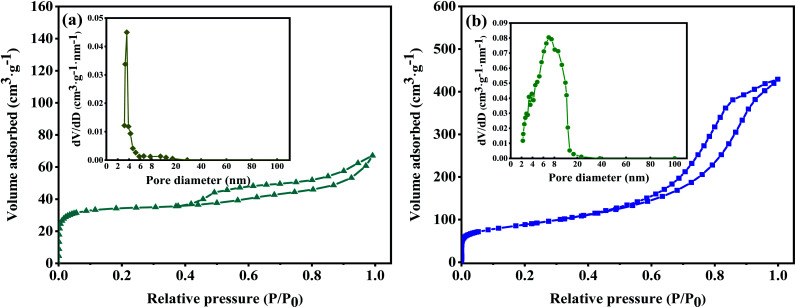
Nitrogen adsorption–desorption isotherms of TiOF_2_ (a) and 10 M-TiOF_2_ (b). The inset shows the corresponding pore size distribution of TiOF_2_ and 10 M-TiOF_2_.

The specific surface area and pore size distribution of TiOF_2_ and 10 M-TiOF_2_ were analysed by nitrogen adsorption–desorption technology. As shown in Fig. 4a, according to the classification of IUPAC, TiOF_2_ shows a typical IV type adsorption desorption isotherm with H4 type hysteresis ring, which represents that the sample has mesopore formation and matches the image presented by Fig. 2a. It can be seen from Fig. 4a inset of BJH pore size distribution diagram that the curves of the TiOF_2_ are sharp, and the pore size distribution is mainly 2–8 nm, which indicates that there is a uniform pore size distribution in catalyst. As shown in Fig. 4b, 10 M-TiOF_2_ exhibit an IV shape of isotherm and an H2b shape hysteresis loop, indicating that there are pipe-shaped pores with uneven pore size distribution and interstitial pores of close-packed spherical particles in the 10 M-TiOF_2_, which is consistent with the results observed in Fig. 2e. The pore size of 10 M-TiOF_2_ is mostly concentrated in 2–20 nm (the inset of Fig. 4b), which indicates that H_2_Ti_3_O_7_ nanorods have ordered mesoporous structure.

The presence of mesopores favours multilight scattering, resulting in enhanced harvesting of the exciting light and thus improved photocatalytic activity.^39,40^ In addition, larger mesopores facilitates faster mass transport, resulting in improved performance.^41–48^

Table 1 shows the specific surface area, pore volume, and average pore size for the tested samples. It can be seen that the specific surface area of 10 M-TiOF_2_ (457 m^2^ g^−1^) is about 114 times that of TiOF_2_ (5 m^2^ g^−1^). After alkali hydrothermal and ion exchange treatment, the specific surface area and pore volume of 10 M-TiOF_2_ were greatly increased compared with TiOF_2_. This indicates that alkali hydrothermal treatment can effectively increase the surface area of the catalyst, which is consistent with the research of Teng *et al.*^49^ 10 M-TiOF_2_ showed a high specific surface area of 457 m^2^ g^−1^ and pore volume of 0.69 cm^3^ g^−1^ which made the TCH adsorb on the surface of the catalyst rapidly. The unsaturated Ti^4+^ in H_2_Ti_3_O_7_ and the delocalized π bond with –OH group in TCH rapidly form ligand to metal charge transfer (LMCT), which greatly improves the response of visible light.^50^ This is confirmed by the subsequent UV-Vis absorbance spectra.

**Table 1:** Specific surface area, pore volume, and average pore size of TiOF_2_ and 10 M-TiOF_2_

Samples	Surface area (m^2^ g^−1^)	Pore volume (cm^3^ g^−1^)	Average pore size (nm)
TiOF_2_	4	0.02	9.02
10 M-TiOF_2_	457	0.69	7.76

### 3.4. FT-IR analysis

**Fig. 5:**
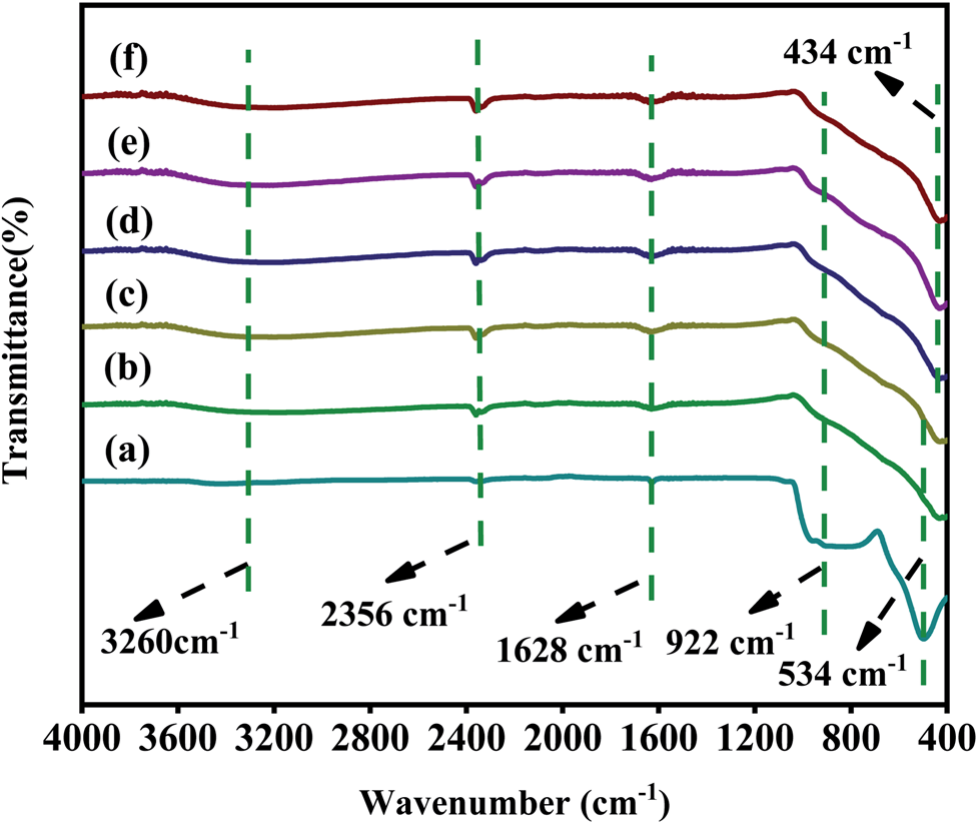
FT-IR spectra of samples: (a) TiOF_2_, (b) 1 M-TiOF_2_, (c) 3 M-TiOF_2_, (d) 5 M-TiOF_2_, (e) 10 M-TiOF_2_ and (f) 15 M-TiOF_2_.

The chemical functional groups of the samples were analysed by FT-IR Fig. 5. The peak at 3260 cm^−1^ corresponds to the O–H stretching vibration of adsorbed water.^51^ The peak at 1628 cm^−1^ corresponds to the bending vibration of Ti–OH, which is attributed to the formation of –OH on the surface of catalysts by hydrothermal process.^52^ The peak at 534 cm^−1^ can be corresponded to the Ti–O vibration in TiOF_2_.^53^ It should be noted that the peak of 1 M-TiOF_2_, 3 M-TiOF_2_, 5 M-TiOF_2_, 10 M-TiOF_2_ and 15 M-TiOF_2_ shifts negatively from 534 cm^−1^ which may be attributed to the increasing number of O_v_ in the lattice structure changes the number of Ti atom surrounding the O atom, and the electron cloud density around a Ti atom decreased.^24^ This was confirmed by EPR and XPS. It can be seen that there are peaks at 922 cm^−1^ in all samples which is attributed to the existence of the Ti–F bond.^54^ Besides, the peak at 2356 cm^−1^ corresponds to the absorbed CO_2_.

### 3.5. Electronic states of photocatalyst

**Fig. 6:**
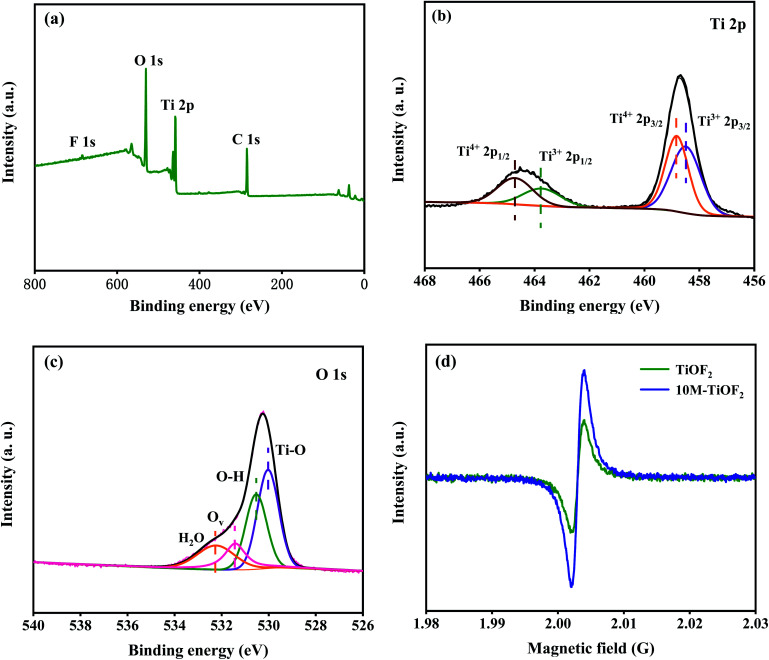
High-resolution XPS spectra XPS spectra of as-prepared 10 M-TiOF_2_: (a) survey spectra. (b) Ti 2p, (c) O 1s; (d) EPR spectra of TiOF_2_ and 10 M-TiOF_2_.

The survey XPS spectra showed Ti, O, C, and F elements in the 10 M-TiOF_2_ (Fig. 6a). The CO_2_ absorbed by the pore structure of the sample leads to the existence of C.^55,56^ Two diffraction peaks at 458.7 and 464.5 eV in the high-resolution spectrum of Ti 2p (Fig. 6b) corresponds to Ti 2p_3/2_ and Ti 2p_1/2_, respectively. The signals at 458.8 and 464.9 eV can correspond to Ti^4+^ 2p_3/2_ and Ti^4+^ 2p_1/2_, and the distance between the two signal peaks is 5.7 eV, which is strong evidence for the existence of Ti^4+^, indicating that a part of Ti in 10 M-TiOF_2_ exists in the form of Ti^4+^.^57,58^ The signal peaks at 458.4 and 463.8 eV can be attributed to the presence of Ti^3+^ 2p_3/2_ and Ti^3+^ 2p_1/2_ in the catalyst. The distance between signal peaks confirmed that a part of Ti existed in the form of Ti^3+^.^33,59^

The states of O in the 10 M-TiOF_2_ were further studied. The spectrum of O 1s orbital is shown in Fig. 6c. The signal peaks at 530.1, 530.5, 531.4, and 532.2 eV is corresponding to the lattice oxygen (Ti–O), hydroxyl oxygen (Ti–OH), oxygen vacancies (O_v_) associated with Ti^3+^, and oxygen in adsorbed water in the catalyst. This indicates that the photocatalyst contains high concentration of Ti^3+^/O_v_. It has been proved in the previous research that oxygen defects are more easily compatible with water molecules in the air and can be further converted into hydroxyl groups, which is consistent with the results of FT-IR. The existence of Ti^3+^/O_v_ species in TiOF_2_ and 10 M-TiOF_2_ was further confirmed by EPR analysis (Fig. 6d). A strong electron paramagnetic signal was observed at *g* = 2.002, which can be attributed to the existence of single electron O_v_ induced by Ti^3+^ in 10 M-TiOF_2_. It is well known that the surface O_v_ associated with Ti^3+^ is extremely unstable in water or air because it is easily oxidized to O_2_^−^.^60^ However, no signal of surface Ti^3+^ species (*g* = 2.02) was observed in EPR spectra. Therefore, Ti^3+^/O_v_ species exist inside the catalyst rather than on the surface, which makes it stable in air or aqueous solution.

### 3.6. Optical performance analysis

**Fig. 7:**
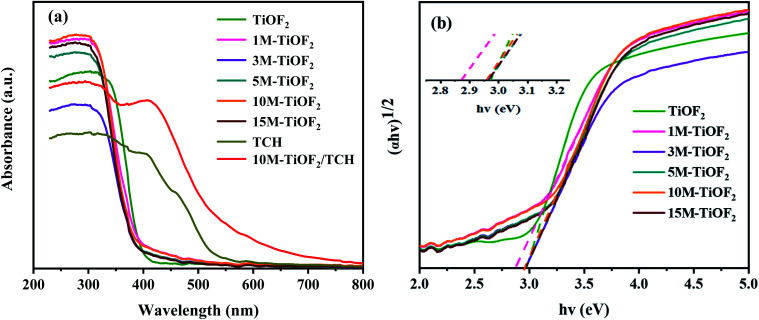
(a) The UV-Vis DRS of prepared samples and (b) the curves of the (*αhv*)^1/2^*versus hv* of prepared samples.

The optical absorption properties of the samples were analysed by UV-Vis-DRS. Fig. 7a shows the light absorption properties of TCH, different photocatalysts, and their complex. In the absence of TCH adsorption, all photocatalysts have strong absorption for the light with a wavelength of less than 390 nm. The amount of light absorbed in the UV region increased in the order 3 M-TiOF_2_ < TiOF_2_ < 5 M-TiOF_2_ < 15 M-TiOF_2_ < 1 M-TiOF_2_ < 10 M-TiOF_2_. However, the photocatalysts exhibit weak absorption bands in the visible region. TCH molecule has a delocalized π bond with the –OH group, which leads to a small energy gap between the highest occupied molecular orbital (HOMO) and the lowest unoccupied molecular orbital (LUMO).^50^ Therefore, TCH has a strong absorption band in the UV and visible regions. Interestingly, the complex of 10 M-TiOF_2_ and TCH showed a stronger absorption band in the visible region than TCH. The complex of 10 M-TiOF_2_ and TCH displayed the strongest absorption band in the visible region.

Most researchers believe that the ligand-to-metal charge transfer (LMCT) between TCH and coordinatively unsaturated Ti(iv) atom is the main reason for the new absorption band and redshift of the complex formed by TCH and Ti-based semiconductor (TiO_2_ and TiOF_2_).^24,61^ H_2_Ti_3_O_7_, as a Ti based semiconductor rich in unsaturated coordination Ti(iv), naturally has an LMCT mechanism with TCH. This may be an important reason for the broadening of the response region of the 10 M-TiOF_2_/TCH to visible light. Besides, TCH adsorbed on H_2_Ti_3_O_7_ but not interacted with Ti(iv) atom, due to its own visible light absorption characteristics, will be similar to the dye sensitization effect to promote the absorption of H_2_Ti_3_O_7_ to visible light. The bandgap energy (*E*_g_) of different photocatalysts was calculated according to the Tauc curve (Fig. 7b). The bandgap energies of TiOF_2_, 1 M-TiOF_2_, 3 M-TiOF_2_, 5 M-TiOF_2_, 10 M-TiOF_2_, and 15 M-TiOF_2_ are 2.97, 2.87, 2.97, 2.97, 2.96, and 2.98 eV. The narrow bandgap of 1 M-TiOF_2_ may be due to the heterojunction formed by cubic TiOF_2_ and H_2_Ti_3_O_7_.

### 3.7. Charge separation properties

**Fig. 8:**
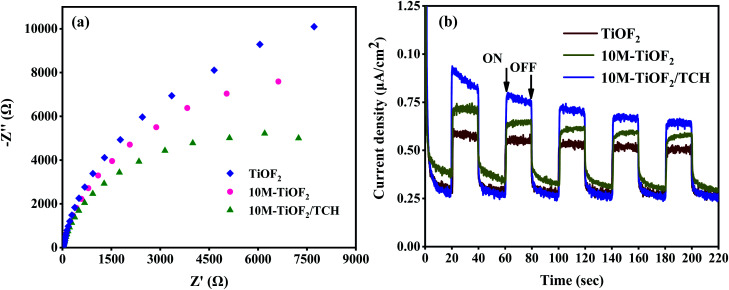
(a) EIS Nyquist plots of TiOF_2_, 10 M-TiOF_2_ and 10 M-TiOF_2_/TCH and (b) transient photocurrent responses of TiOF_2_, 10 M-TiOF_2_ and 10 M-TiOF_2_/TCH.

EIS is an effective technique to study interface charge separation and transfer.^62^ The EIS Nyquist plots were performed to assess the conductivity of different samples. As shown in Fig. 8a, the semicircle diameter of TiOF_2_ is larger than that of 10 M-TiOF_2_, which indicates that 10 M-TiOF_2_ has a lower electron transfer resistance. This may be because the band gap of 10 M-TiOF_2_ is larger than that of TiOF_2_, resulting in a higher carrier recombination degree than TiOF_2_. The arc radius of 10 M-TiOF_2_/TCH is smaller than that of TiOF_2_ and 10 M-TiOF_2_, indicating that photogenerated electrons of 10 M-TiOF_2_/TCH transfer fastest. This might be due to the LMCT effect between 10 M-TiOF_2_ and TCH, which makes the complex have smaller internal resistance and promotes the transfer efficiency of interface electrons. Moreover, under periodic sunlight, the photocurrent–time (*I*–*t*) curves of the synthesized TiOF_2_, 10 M-TiOF_2_, and 10 M-TiOF_2_/TCH are shown in Fig. 8b. Obviously, the TiOF_2_ and 10 M-TiOF_2_ electrodes exhibit relatively low current intensity with a certain attenuation, which strongly reveals the internal recombination of light-induced charge carriers. When TCH is attached to the surface of 10 M-TiOF_2_, the photocurrent intensity is significantly increased, which means that the LMCT mechanism formed when TCH is adsorbed on the surface of 10 M-TiOF_2_ can effectively promote the rapid transfer of charge carriers, thereby enhancing the photocatalytic activity. This result is similar to that of Zhang *et al.*^61^

### 3.8. Photocatalytic performance

TCH is an extremely stable organic compound, which seldom self-degrades under light. Hence, the degradation of TCH must take place with the help of photocatalyst. This has been confirmed in previous studies.^4,10^

**Fig. 9:**
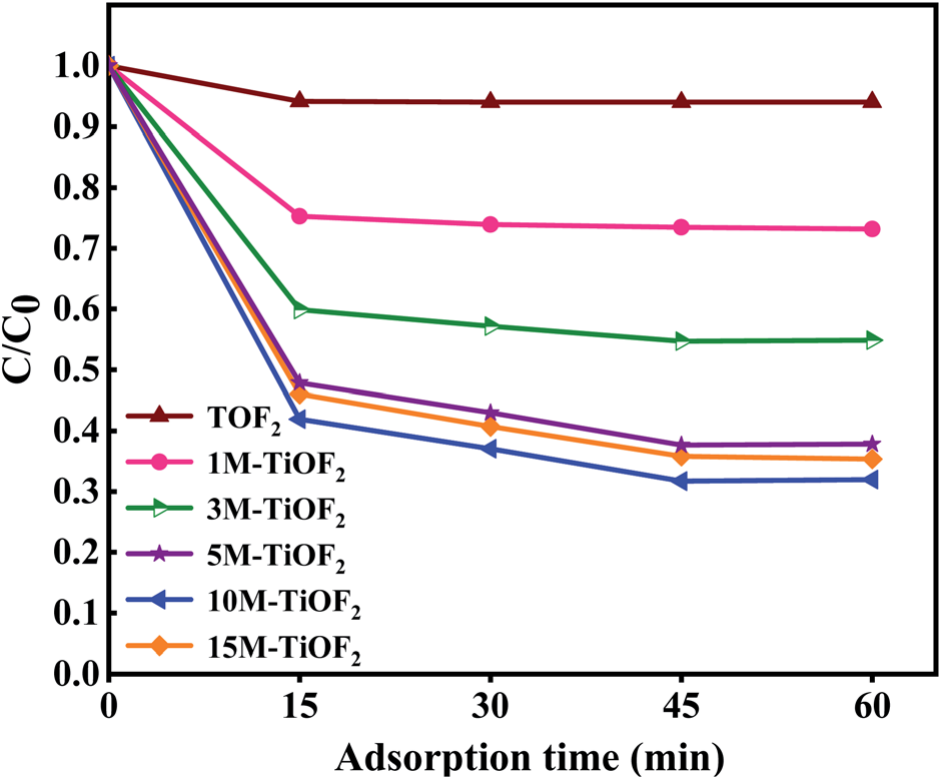
Adsorption of TCH solution (20 mg L^−1^, 100 mL) using different photocatalysts (0.2 g L^−1^) in dark for 60 min.

The adsorption degradation performance of different photocatalysts for TCH solution is shown in Fig. 9. It can be seen that different photocatalysts have reached adsorption–desorption equilibrium for TCH at 60 min. For TiOF_2_, 1 M-TiOF_2_, 3 M-TiOF_2_, 5 M-TiOF_2_, 10 M-TiOF_2_, and 15 M-TiOF_2_, when the adsorption–desorption reach equilibrium, the removal rates of TCH were 6%, 27%, 45%, 62%, 68%, and 65%, respectively.

The photocatalytic degradation performance of different photocatalysts for TCH solution is shown in Fig. 9a. Without the addition of photocatalyst, TCH almost has no self-degradation under simulated sunlight. After the simulated sunlight was turned on, the total removal rate of TCH by TiOF_2_ was only 10%, which indicated that TiOF_2_ showed the worst photocatalytic activity. Surprisingly, the removal rate of TCH by 10 M-TiOF_2_ reached 88% only after 20 min illumination under simulated sunlight. After further illumination for 60 min, the total removal rate reached 92%. 1 M-TiOF_2_, 3 M-TiOF_2_, 5 M-TiOF_2_, and 15 M-TiOF_2_ also had higher removal rates of TCH, which were 56%, 88%, and 90%.

**Fig. 10:**
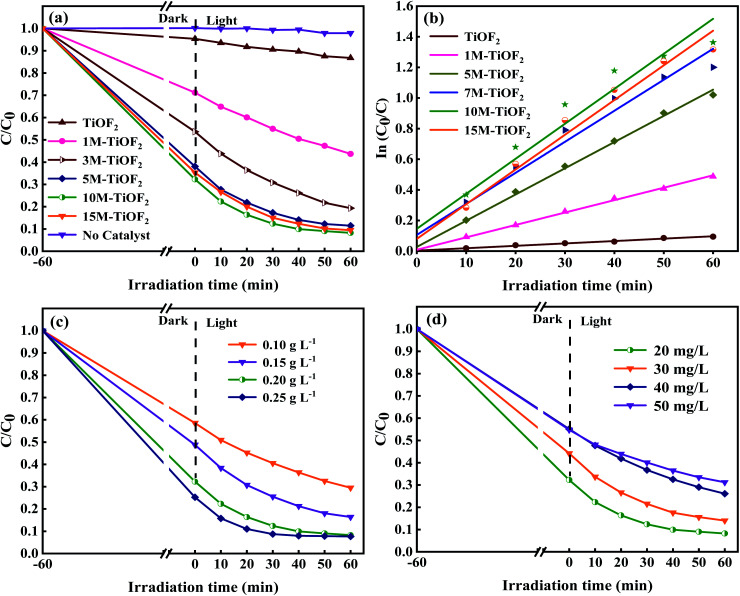
(a) Photodegradation of TCH solution using different photocatalysts under simulated sunlight; (b) kinetics for as-prepared samples; (c) the effect of different dosage of 10 M-TiOF_2_ on the degradation of TCH solution; (d) the effect of different concentration of TCH solution on the photocatalytic performance of 10 M-TiOF_2_ under simulated sunlight.

Furthermore, the exposure of {104} facets make H_2_Ti_3_O_7_ have high reactivity, and the synergistic effect of LMCT and dye sensitization enhances the visible light absorption of the photocatalyst. Fig. 10b shows the kinetic curves of photocatalysts. Typically, the variation of TCH concentration on irradiation time under simulated solar irradiation matches the pseudo first-order kinetic model.^63^ The corresponding apparent rate constants *k* of TiOF_2_, 1 M-TiOF_2_, 3 M-TiOF_2_, 5 M-TiOF_2_, 10 M-TiOF_2_, and 15 M-TiOF_2_ were calculated to be 0.00156, 0.00811, 0.01711, 0.02026, 0.02285 and 0.02267 min^−1^, respectively. The photocatalytic degradation rate of 10 M-TiOF_2_ is about 15 times that of the TiOF_2_.

**Fig. 11:**
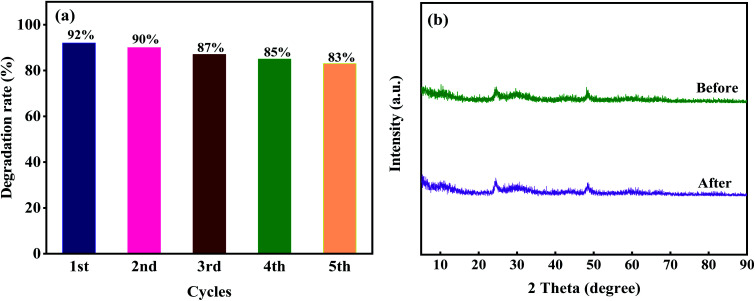
(a) Stability tests over 10 M-TiOF_2_ for TCH degradation; (b) XRD patterns of 10 M-TiOF_2_ before and after five cycling runs.

The effects of different dosage of 10M-TiOF_2_ (0.1 g L^−1^, 0.2 g L^−1^, 0.3 g L^−1^, 0.4 g L^−1^) on photocatalytic degradation of TCH were investigated (Fig. 10c). Photocatalyst was 0.1 g L^−1^, the degradation efficiency was only 71%, which might be due to the fact that less photocatalyst is not enough to produce more active sites in the reaction system. When the dosage of 10M-TiOF_2_ catalyst is 0.15 g L^−1^, the degradation rate of TCH was 84%. Furthermore, the addition of 0.2 g L^−1^ and 0.25 g L^−1^ of catalyst can be as high as 92%. In addition, the effects of different concentrations of TCH solution on the photocatalytic performance of 10 M-TiOF_2_ sample were studied (Fig. 10d). Obviously, 10 M-TiOF_2_ had the best degradation effect on TCH solution of 20 mg L^−1^. With the increase of TCH solution concentration, the photocatalytic performance of 10 M-TiOF_2_ gradually decreased. It may be that the photocatalyst is not to provide enough active reaction sites to deal with so many TCH molecules.^24^ Furthermore, the repeatability and sedimentation performance of the 10 M-TiOF_2_ were evaluated. Fig. 11a shows the results of five consecutive cycles of photocatalytic degradation experiments. After five cycles, the degradation rate of 10 M-TiOF_2_ decreased from 92% to 83%, indicating that 10 M-TiOF_2_ has a high reuse rate. Fig. 11b shows the XRD pattern of 10 M-TiOF_2_ after five cycles. The phase structure of the photocatalyst did not change obviously before and after the reaction, which indicated that the photocatalyst had good structural stability and reusability.

### 3.9. Photocatalytic mechanism

**Fig. 12:**
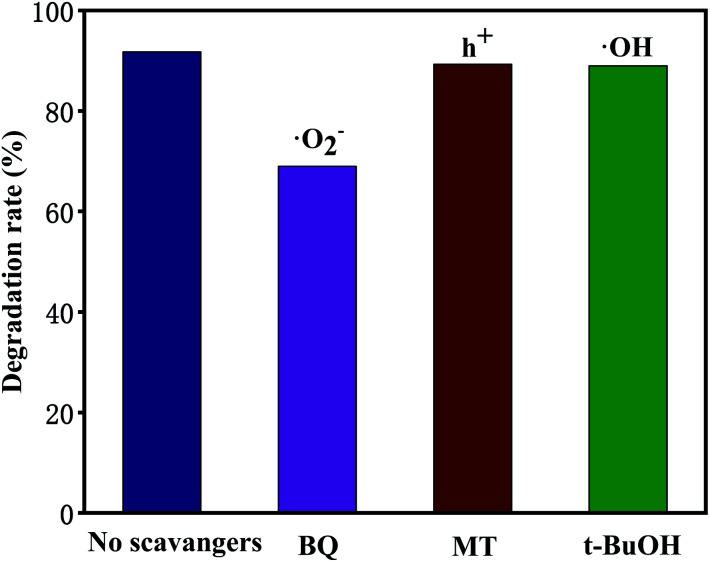
Photocatalytic degradation rate of 10 M-TiOF_2_ with the addition of different scavengers.

Trapping tests were carried out to examine the photocatalytic mechanism of 10 M-TiOF_2_ (Fig. 12). Scavengers such as BQ, MT, and *t*-BuOH were employed to capture ˙O_2_^−^, h^+^, and ˙OH species, respectively.^6,37^ The removal rate of TCH decreased from 92% to 69% when BQ was added into the system, which indicated that ˙O_2_^−^ was the main active species in the degradation system. After adding MT and *t*-BuOH, the photocatalytic degradation was almost not inhibited, indicating that h^+^ and ˙OH did not play a major role in photodegradation.

**Fig. 13:**
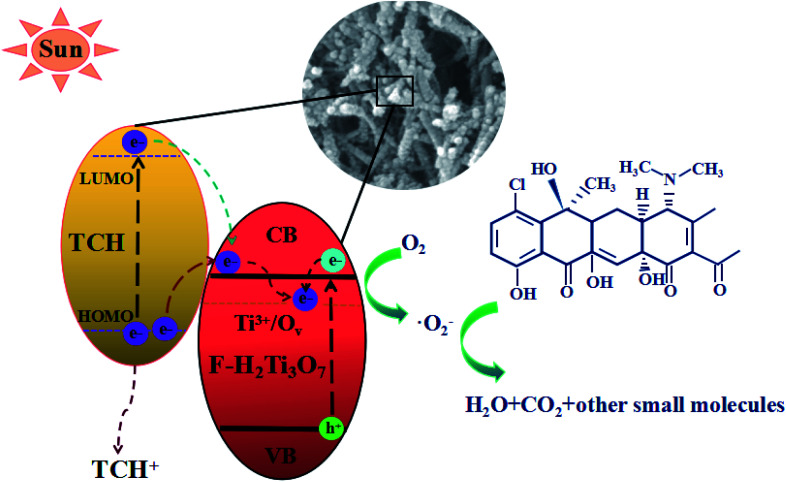
Schematic shows the TCH degradation by 10 M-TiOF_2_ photocatalyst.

As shown in Fig. 13, derived from the analysis presented above, we put forward the possible mechanism of degradation of TCH by 10 M-TiOF_2_ under simulated sunlight. Firstly, the {104} facets of H_2_Ti_3_O_7_ nanorods has a strong adsorption capacity for TCH. Furthermore, the π orbital of TCH may form electronic coupling with the 3d orbital of Ti^4+^, resulting in a surface complex between TCH and H_2_Ti_3_O_7_.^27^ TCH was degraded simultaneously in two pathway. In the first path, electrons are excited from HOMO level of TCH to LUMO level under simulated sunlight, and then rapidly injected into the conduction band (CB) position of H_2_Ti_3_O_7_ facets.^24^ However, TCH lost its electrons and remained a stable product (TCH^+^). Such photoexcitation and injection process is often referred to as LMCT. In the second path, because of the limited number of unsaturated Ti(iv) atoms, the electrons of another part of uncoordinated TCH transition from the ground state (HOMO) to the excited state (LUMO), leaving a TCH^+^ and further transfer to the conduction band of F-H_2_Ti_3_O_7_ under the excitation of simulated sunlight. This process of light excitation and electron transfer is usually called dye sensitization. For F-H_2_Ti_3_O_7_, Ti^3+^/O_v_ are widely distributed on the surface, which means that there are abundant traps on the surface. Due to the delocalization of electrons in the CB, the electrons transferred from TCH to F-H_2_Ti_3_O_7_ CB and the electrons transferred from F-H_2_Ti_3_O_7_ VB can easily migrate from F-H_2_Ti_3_O_7_ CB to the surface trap, where they are trapped and stabilized.^64,65^ Due to the CB potential, F-H_2_Ti_3_O_7_ is more negative than that of O_2_/˙O (−0.046 eV *vs.* NHE),^66^ the adsorbed O_2_ on the surface of the photocatalyst is more likely to capture the electrons and form ˙O_2_^−^. These reactive superoxide radicals (˙O_2_^−^) can highly oxidize the TCH and TCH^+^ into H_2_O, CO_2_, and some small molecules.

## 4. Conclusions

In summary, F-doped H_2_Ti_3_O_7_-{104} nanorods with oxygen vacancies can be prepared by alkaline hydrothermal method combined with ion-exchange method using TiOF_2_ as precursor. TiOF_2_/H_2_Ti_3_O_7_ composite can be formed in the low concentration KOH solution (1 mol L^−1^), and H_2_Ti_3_O_7_ single phase can be formed in high concentration KOH solution (>3 mol L^−1^). The synergy of LMCT effect between TCH and H_2_Ti_3_O_7_ not only expands the visible light absorption region of H_2_Ti_3_O_7_, but also improves the carrier separation ability. Thereby, it has excellent photocatalytic degradation performance for TCH. Furthermore, F-doped H_2_Ti_3_O_7_-{104} nanorods stronger photochemical corrosion resistance. This simple and low-cost strategy can be used for large-scale production of photocatalysts, which is conducive to the practical application of wastewater purification process.

## Conflicts of interest

The authors declare that they have no known competing financial interests or personal relationships that could have appeared to influence the work reported in this paper.

## Supplementary Material
